# Systemic Inflammation and its association with functional impairment and mortality in patients with disease-related malnutrition: findings from the AFEDIN cohort

**DOI:** 10.3389/fnut.2026.1771862

**Published:** 2026-05-14

**Authors:** Alfonso Vidal-Casariego, Samara Palma Milla, Juan José López Gómez, José Manuel García Almeida, Isabel Vegas Aguilar, Juan Manuel Guardia Baena, Cristina Porca Fernández, Patricia Mezzerhane Riccardi, Agustín Ramos Prol, Pedro Pablo García Luna, Patricia Pérez Castro, Natalia C. Iglesias Hernández, Luis M. Luengo Pérez, Daniel de Luis Román

**Affiliations:** 1 Servicio de Endocrinología y Nutrición, Complexo Hospitalario Universitario de A Coruña, A Coruña, Spain; 2 Servicio de Endocrinología y Nutrición, Hospital Universitario La Paz, Madrid, Spain; 3 Servicio de Endocrinología y Nutrición, Hospital Clínico Universitario de Valladolid, Centro de Investigación de Endocrinología y Nutrición Facultad Medicina, Health Research Institute of Valladolid (IBioVALL), Valladolid, Spain; 4 Servicio de Endocrinología y Nutrición, Hospital Virgen de la Victoria, Málaga, Spain; 5 Servicio de Endocrinología y Nutrición, Hospital Universitario Virgen de las Nieves, Granada, Spain; 6 Servicio de Endocrinología y Nutrición, Complexo Hospitalario Universitario de Ferrol, Ferrol, Spain; 7 Servicio de Endocrinología y Nutrición, Hospital Universitario Marqués de Valdecilla, Santander, Spain; 8 Servicio de Endocrinología y Nutrición, Hospital Universitario La Fe, València, Spain; 9 Servicio de Endocrinología y Nutrición, Hospital Universitario Virgen del Rocío, Sevilla, Spain; 10 Servicio de Endocrinología y Nutrición, Hospital Universitario Álvaro Cunqueiro, Vigo, Spain; 11 Servicio de Endocrinología y Nutrición, Hospital Universitario de Basurto, Bilbao, Spain; 12 Servicio de Endocrinología y Nutrición, Hospital Universitario de Badajoz, Badajoz, Spain

**Keywords:** c-reactive protein, functional status, inflammation, malnutrition, mortality

## Abstract

**Introduction:**

The Global Leadership Initiative on Malnutrition (GLIM) framework incorporates inflammation as a key etiologic criterion for diagnosing disease-related malnutrition (DRM), yet the clinical implications of systemic inflammation, particularly as quantified by C-reactive protein (CRP), remain insufficiently characterized.

**Methods:**

To evaluate whether CRP-based inflammation thresholds proposed by GLIM are associated with impaired nutritional status, diminished functional capacity, reduced quality of life, and increased short-term mortality in ambulatory patients with DRM. This prospective, multicenter cohort study (AFEDIN – Analysis of the Etiological Factors of Malnutrition: Inflammation and Intake) included 266 ambulatory patients with DRM and CRP > 3 mg/L. Participants underwent comprehensive nutritional assessment via anthropometry, bioelectrical impedance, and nutritional ultrasound, alongside functional testing (handgrip strength, Timed Up and Go, chair stand test), quality of life evaluation (EQ-5D-5L), and inflammatory profiling. Patients were stratified by CRP levels into mild (3–9.9 mg/L), moderate (10–49.9 mg/L), and severe (≥50 mg/L) inflammation groups.

**Results:**

C-reactive protein levels were strongly associated with inflammatory and biochemical markers but not with direct measures of body composition. Higher CRP concentrations correlated with reduced performance on functional tests and increased difficulty with self-care. Mortality increased markedly across CRP strata (1.1%, 11.8%, and 17.7%, respectively; *p* < 0.001). Compared to patients with CRP levels of 3–9.9 mg/L, those with CRP ≥ 50 mg/L had a significantly higher risk of mortality (OR 20.14, 95% CI 2.47–164.1, *p* < 0.05), as did those with CRP 10–49.9 mg/L (OR 12.53, 95% CI 1.62–97.1, *p* < 0.05).

**Conclusion:**

In ambulatory DRM patients, higher CRP levels predict functional impairment and mortality, highlighting the prognostic utility of CRP within the GLIM framework. Inflammation should be routinely quantified to enhance risk stratification and guide intervention.

## 1 Introduction

Malnutrition is defined as a clinical condition resulting from reduced nutrient intake or absorption, leading to alterations in body composition (particularly fat-free mass), reduced body cell mass, and impaired functional capacity (1). The causes of malnutrition are highly variable, including inadequate intake, increased losses, or elevated acute or chronic energy–protein requirements, associated or not with disease.

The term disease-related malnutrition (DRM) was introduced in 2010 to highlight the impact of inflammation on nutritional status (2). Accordingly, an etiological classification was proposed based on the presence or absence of inflammation and its severity (3). This framework distinguishes between malnutrition caused purely by starvation, where inflammation is absent, and DRM, where an inflammatory component–acute or chronic–plays a central role in disrupting normal metabolism and nutrient utilization. Inflammation contributes to increased catabolism, reduced anabolism, and alterations in hormonal and cytokine responses that collectively exacerbate muscle loss and impair tissue repair. In the acute setting, such as in sepsis or major surgery, the inflammatory response can be intense and short-lived, while in chronic diseases like cancer or heart failure, sustained but lower-grade inflammation predominates, leading to more progressive nutritional deterioration. This etiological classification underscores the need to tailor nutritional interventions according to the underlying pathophysiology, considering not only the degree of weight loss and muscle wasting but also the inflammatory environment that modulates the patient’s metabolic demands and response to nutritional support (4).

The inflammatory response triggered by disease or injury is accompanied by metabolic alterations, including increased energy expenditure and nitrogen excretion, leading to elevated protein requirements. It also contributes to insulin resistance, suppression of appetite (disease-related anorexia), and enhanced muscle catabolism, among other effects. The presence of this inflammatory component may compromise the effectiveness of medical, surgical, or nutritional interventions, potentially explaining the higher morbidity, mortality, rates of readmission, and health costs attributed to hospital malnutrition (5–7).

In 2018, the Global Leadership Initiative on Malnutrition (GLIM) criteria were published through a consensus of the European Society for Clinical Nutrition and Metabolism (ESPEN), the American Society for Parenteral and Enteral Nutrition (ASPEN), the Asian PENSA, and the Latin American FELANPE societies (8). Based on a positive nutritional screening tool, the proposed diagnostic algorithm incorporates phenotypic criteria (body weight loss, low BMI, and reduced muscle mass) with severity thresholds, together with etiologic criteria (reduced intake or absorption, and the presence of an inflammatory component). Malnutrition is diagnosed when at least one phenotypic criterion and one etiologic criterion are met.

The etiologic criterion of inflammation is generally established according to the underlying disease context. For example, cancer is considered a prototypical chronic inflammatory condition, whereas major burns represent an acute and severe inflammatory example. A recent review of 79 studies applying the GLIM framework showed that 85% of studies identified the etiologic inflammation criterion based on the patient’s underlying disease, while only 25% included inflammatory biomarkers such as C-reactive protein (CRP), and 7% did not specify the basis for fulfilling this criterion (9). In response to these gaps, an international panel has recently developed specific recommendations to guide the practical assessment of inflammation within the GLIM framework. These recommendations, based on a modified Delphi consensus, emphasize the integration of clinical judgment, the underlying disease diagnosis, observable clinical signs, and CRP levels whenever available, to confirm the inflammatory component. Pragmatic CRP cut-off points were proposed, with values of 3–9.9 mg/L for mild inflammation, 10–50 mg/L for moderate inflammation, and above 50 mg/L for severe inflammation, while also recognizing the complementary role of other laboratory markers or clinical indicators (10).

The objective of this study, conducted within the framework of the AFEDIN study (Análisis Daniel de Luis Román los Factores Etiológicos de Desnutrición: inflamación e ingesta – Analysis of the Etiological Factors of Malnutrition: Inflammation and Intake), was to evaluate whether the inflammatory criterion proposed by GLIM–specifically, the use of C-reactive protein (CRP) thresholds as suggested in ESPEN-endorsed guidance–is associated with poorer nutritional status (assessed by anthropometry, bioelectrical impedance analysis, and nutritional ultrasound), reduced functional capacity, diminished quality of life, and higher mortality in patients diagnosed with DRM.

## 2 Patients and methods

### 2.1 Study design

This was a prospective, multicenter, observational, cross-sectional analysis derived from a prospective multicenter cohort study conducted in clinical nutrition units across multiple Spanish hospitals. All clinical assessments were performed during the initial visit, once the patient met inclusion criteria and signed informed consent. Evaluations were conducted by trained personnel using standardized procedures. The present study was registered in ClinicalTrials.gov (NCT05781178).

### 2.2 Study population

Patients were recruited consecutively from clinical nutrition units across participating Spanish hospitals between July 2023 and July 2024.

Inclusion criteria were male or female ambulatory participants aged over 18 years; diagnosis of DRM, whether acute or chronic; presence of an inflammatory response, defined as CRP > 3 mg/L; voluntary agreement to participate in the study after reading the participant information sheet and signing informed consent; and sufficient educational level and cognitive capacity to understand the study procedures.

Exclusion criteria included: pregnancy or breastfeeding; advanced liver disease, including cirrhosis or chronic hepatitis classified as Child-Pugh C; advanced neoplastic disease with an expected survival of less than 6 months; renal insufficiency with a creatinine clearance below 45 mL/min; severe infection requiring hospitalization within the past 3 weeks; current antibiotic treatment, except for prophylactic use; current or recent (within 1 month) use of corticosteroids or biological therapies (e.g., monoclonal antibodies); chronic use of non-steroidal anti-inflammatory drugs (NSAIDs); current intake of omega-3 supplements for any comorbid condition; undergoing surgery during the study follow-up period; development of an infectious condition during follow-up; and initiation of any medication during the study period deemed by the investigator to potentially alter the patient’s inflammatory status. In addition, patients were excluded if they presented any condition that, in the opinion of the principal investigator, could interfere with study conduct. This criterion included situations such as severe cognitive impairment precluding informed consent or valid completion of study assessments, anticipated relocation preventing follow-up, inability to comply with study procedures, or acute clinical instability not otherwise covered by the predefined exclusion criteria.

### 2.3 Biochemical and inflammatory markers

Blood samples were obtained at baseline and analyzed in the clinical laboratory of each participating center. Given the multicenter design of the study, no centralized laboratory was used. All participating laboratories are accredited hospital laboratories operating under standardized analytical protocols, with internal and external quality control procedures in accordance with national regulatory standards. Serum C-reactive protein (CRP) was measured using standardized immunoturbidimetric or high-sensitivity immunoassay methods routinely implemented in each laboratory. Serum albumin was determined using automated colorimetric methods (e.g., bromocresol green), and prealbumin by immunonephelometry or immunoturbidimetry, according to local laboratory procedures.

Inflammatory indices such as the CRP/albumin ratio, CRP/prealbumin ratio, neutrophil-to-lymphocyte ratio, platelet-to-lymphocyte ratio, and systemic immune-inflammation index were calculated from the corresponding laboratory values.

### 2.4 Nutritional assessment

Nutritional risk was screened using the Malnutrition Universal Screening Tool (MUST). Diagnosis of malnutrition was established based on GLIM criteria, requiring at least one phenotypic and one etiological criterion (including CRP > 3 mg/dL).

Anthropometric measurements were performed with patients in light clothing and without shoes. Body weight and height were measured by calibrated scales and stadiometers. Body mass index (BMI) was calculated as body weight (kg)/height (m^2^). Calf Circumference was measured at the point of maximum circumference of the non-dominant leg, with the patient seated and the knee at a 90° angle. Dietary intake was assessed at baseline using a 48-h dietary recall administered by trained personnel. Nutrient composition was analyzed using the online diet calculator provided by the Centro de Investigación de Endocrinología y Nutrición Clínica (IENVA)^1^, which utilizes comprehensive Spanish food composition tables.

Body composition was assessed using single frequency bioimpedance (50 kHz) obtaining resistance, reactance, and phase angle values. Based on the aforementioned values, the appendicular skeletal muscle index (ASMI), fat mass (FM), and fat-free mass index (FFMI), as well as body cell mass (BCM) and body water (extra- and intracellular), were obtained using the AKERN NutriLab^®^ (Akern S.L., Pisa, Italy). Bioimpedance was performed with the subject in a supine position on a non-conductive surface, with the limbs abducted at 45°. Fasting for more than 2 h was recommended, avoiding the intake of alcohol, coffee, and caffeinated soft drinks in the previous 24 h, as well as vigorous exercise.

Nutritional ultrasound^®^ protocol was applied to assess body composition (11). Assessment was performed with a high-frequency linear transducer to evaluate quadriceps rectus femoris (width, thickness, and cross-sectional area) of the dominant lower extremity, with a 10–12 MHz probe (Mindray Z60, Madrid, Spain). Measurements were taken from the anterior thigh while the patient lay supine with knees fully extended and relaxed. The imaging site was standardized to two-thirds of the femur’s length, measured from the anterior superior iliac spine to the upper border of the patella. Parameters for muscle mass included the rectus femoris muscle area, rectus femoris muscle thickness and width, corresponding to the cross-sectional area and thickness of the muscle belly. Subcutaneous adipose tissue (superficial and deep layers) and preperitoneal fat were measured at the midpoint between the xiphoid process and the umbilicus.

### 2.5 Functional evaluation

Functional status was assessed using three validated tools. Handgrip strength was measured with a Jamar^®^ (Sammons Preston, Bolingbrook, IL, USA), hydraulic dynamometer on the dominant hand. The mean value of three trials, with 30-s rests between attempts, was recorded. The Timed Up and Go (TUG) Test records the time (in seconds) required for a patient to rise from a standard chair, walk 3 m, turn around, return, and sit back down. A time ≥ 20 s was considered indicative of impaired mobility and high risk of falls; values < 10 s were considered normal functional mobility. Intermediate values (10–19 s) were interpreted as showing some degree of functional limitation depending on patient context. The Chair Stand Test measures the number of full stands a patient can perform from a seated position in 15 s (arms crossed over the chest).

### 2.6 Quality of life

Assessed with the EQ-5D-5L questionnaire, including both the five-dimension index and the EQ visual analogue scale (EQ-VAS). The questionnaire describes health status in terms of five dimensions: mobility, self-care, daily activities, pain or discomfort, and anxiety or depression. Each of these dimensions is divided into three levels of severity (no problems, some problems, and extreme problems). These data are later converted into a single general score (EQ-5D index) using a predefined value table. The index ranges from 1 (best health status) to 0 (worst health status).

### 2.7 Ethical considerations

All participants received written and verbal information regarding the study objectives, procedures, and potential risks and benefits. Informed consent was obtained from all patients prior to any study-related activities. Participation was voluntary, and patients were free to withdraw at any point without affecting their standard medical care. Data confidentiality and patient anonymity were preserved throughout the study in compliance with the European General Data Protection Regulation (GDPR) 2016/679 and Spanish Organic Law 3/2018 on Personal Data Protection and Digital Rights.

### 2.8 Statistical analysis

Continuous variables were presented as means and standard deviations (SD) or medians and interquartile ranges (IQR), depending on data distribution assessed by the Shapiro–Wilk test. Categorical variables were expressed as frequencies and percentages. Comparisons between CRP-based inflammation strata were performed using the chi-square test for categorical variables, and ANOVA or Kruskall-Wallis test for continuous variables, as appropriate. The relationship between inflammation categories and 3-months mortality was assessed using Kaplan–Meier survival curves and the log-rank test. Cox proportional hazards regression was applied to estimate hazard ratios (HR) and 95% confidence intervals (CI), adjusting for relevant covariates such as age, sex, malnutrition severity (GLIM stage), and handgrip strength. Statistical significance was defined as *p* < 0.05.

## 3 Results

### 3.1 Descriptive characteristics of the study population

Of the 266 patients included in the study, 208 (78.2%) attended the final (V3) visit, whereas 28 (10.5%) did not attend and 30 (11.3%) had missing final visit data. Reported reasons for non-attendance included voluntary withdrawal, relocation outside the study area, clinical deterioration requiring hospitalization or palliative care, and loss to follow-up. The mean age was 66.6 years (SD: 12.9; range: 21–95) and there was predominance of males (61.7%). Regarding nutritional status, 89.1% of patients had a MUST score ≥ 2, and 49.2% met GLIM criteria for moderate and 50.8% for severe DRM. Functional capacity was reduced in a considerable proportion of the sample: 22.2% had a TUG > 20 s, 41.8% performed fewer than five repetitions on the chair stand test, and 55.0% presented low handgrip strength based on sex-specific reference values. The mean CRP concentration in the study population was 32.4 mg/L (SD: 42.8). When stratified by CRP values, 35.8% of patients had levels between 3 and 9.9 mg/L, 44.9% between 10 and 49.9 mg/L, and 19.3% presented with severe inflammation, defined as CRP ≥ 50 mg/L. Main characteristics are summarized in Table 1.

**TABLE 1:** Baseline characteristics of the study population by CRP group.

Variable		10–49.9 mg/L	≥50 mg/L	*P*-value
Age (years)	65.4 (12.6)	68.02 (13.2)	65.2 (12.7)	0.244
Male sex (%)	54.7	59.7	78.7	0.013
Primary diagnosis (%)				
Cancer	56.8	78.2	78.4	0.510
Digestive diseases	12.6	7.6	3.9	0.859
Surgery	6.3	0.8	11.8	1.000
Other	21.1	12.6	5.9	0.945
Neurological conditions	3.2	0.8	0.0	–
Comorbidities (%)				
Hypertension	35.8	38.6	41.2	0.807
Diabetes mellitus	18.9	19.3	17.7	1.000
COPD	14.7	10.9	3.9	0.123
CKD	1.1	1.7	0.0	1.000
Heart failure	2.1	4.2	5.9	0.435
MUST ≥ 2 (%)	85.1	90.8	94.1	0.228
Sarcopenia (%)	65.0	76.5	74.5	0.222
Severe malnutrition (%)	42.5	50.0	58.5	0.437
Weight (kg)	58.6 (13.4)*	64.0 (18.5)	66.2 (13.1)*	0.009
BMI (kg/m^2^)	21.9 (4.5)*	23.4 (6.0)	23.7 (3.9)*	0.047
Body weight loss (%)	12.2 (10.6)	13.7 (9.5)	12.6 (11.1)	0.555
Calf circumference (cm)	31.3 (5.1)	32.5 (4.9)	33.3 (3.4)	0.049

Values are expressed as mean (standard deviation) or percentage, as indicated. BMI, body mass index; COPD, chronic obstructive pulmonary disease; CKD, chronic kidney disease; MUST, Malnutrition Universal Screening Tool. For the comparison among groups *p < 0.05.

### 3.2 Dietary intake

Mean daily energy intake was 29.1 (11.2) kcal/kg overall, with no significant differences across CRP strata: 30.1 (11.5) kcal/kg in the 3–9.9 mg/L group, 33.1 (21.7) kcal/kg in the 10–49.9 mg/L group, and 27.7 (18.9) kcal/kg in the ≥50 mg/L group (*p* = 0.357). Mean daily protein intake was 1 (0.5) g/kg, without differences across groups: 1.99 (5.57) g/kg in the 3–9.9 mg/L group, 1.45 (0.82) g/kg in the 10–49.9 mg/L group, and 1.21 (0.63) g/kg in the ≥50 mg/L group (*p* = 0.264) (*p* = 0.127). Regarding omega-3 fatty acids, EPA intake was significantly higher in the intermediate CRP group [238.5 (540.3) mg] compared to the lowest [51.0 (179.2) mg] and highest CRP groups [83.9 (215.1) mg], with *p* = 0.004. DHA intake followed a similar pattern, at 163.7 (338.1) mg in the intermediate group, compared to 45.9 (123.2) mg in the lowest and 78.8 (153.1) mg in the highest CRP group (*p* = 0.006). No significant differences were observed for monounsaturated fatty acids, polyunsaturated fatty acids, or cholesterol intake.

### 3.3 Nutritional assessment

Statistically significant differences between CRP strata were observed for reactance and body water compartments. Reactance values decreased progressively with increasing CRP levels, whereas total body water, intracellular water, and extracellular water increased across inflammation categories, indicating a negative association between CRP and reactance and a positive association with body water parameters. Detailed results are presented in Table 2.

**TABLE 2:** Comparison of bioelectrical impedance and nutritional ultrasound parameters by CRP group.

Parameter		10–49.9 mg/L	≥50 mg/L	*P*-value
Resistance (Ω)	564.3 (117.9)	545.2 (105.3)	532.3 (107.4)	0.264
Reactance (Ω)	49.3 (11.7)	46.4 (11.3)	43.4 (8.5)	0.017
Phase angle (°)	4.6 (0.9)	4.6 (0.9)	4.8 (1.2)	0.477
TBW (L)	33.9 (7.2)*	35.4 (8.1)	37.9 (7.3)*	0.011
Intracellular water (L)	16.2 (3.8)	16.9 (4.6)	18.4 (5.4)	0.024
Extracellular water (L)	17.6 (4.7)*	18.5 (4.5)	19.6 (3.6)*	0.041
ECW/TBW ratio	0.52 (0.07)	0.52 (0.06)	0.52 (0.07)	0.794
Fat mass (%)	22.8 (9.7)	24.3 (9.7)	22.4 (9.4)	0.383
FFM index (kg/m^2^)	16.7 (2.9)*	17.1 (3.2)	18.0 (2.8)*	0.034
SMI (kg/m^2^)	2.34 (0.40)*	2.31 (0.42)	2.41 (0.34)*	0.209
ASMI (kg/m^2^)	6.3 (1.5)	6.4 (1.5)	6.8 (1.2)	0.052
Body Cell Mass (kg)	21.5 (5.3)	22.2 (6.3)	24.4 (7.8)	0.032
Subcutaneous abdominal fat (cm)	2.99 (4.07)	2.14 (3.22)	1.81 (1.90)	0.187
Superficial layer (cm)	1.19 (1.41)	0.96 (1.20)	1.92 (5.28)	0.186
Preperitoneal fat (cm)	0.75 (0.78)	0.67 (0.93)	0.64 (0.45)	0.186
Rectus femoris – Y axis (cm)	0.88 (0.37)	1.00 (0.32)	0.94 (0.37)	0.039
Rectus femoris – X axis (cm)	3.32 (0.72)	3.46 (0.72)	3.58 (0.36)	0.105
Rectus femoris area (cm^2^)	3.02 (1.14)	3.18 (1.06)	3.27 (1.14)	0.455

Values are expressed as mean (standard deviation). TBW, total body water; ECW, extracellular water; FFM, fat-free mass; SMI, skeletal muscle index; ASMI, appendicular skeletal muscle index; BCM, body cell mass. For the comparison among groups *p < 0.05.

### 3.4 Functional assessment

Handgrip strength showed no statistically significant differences across CRP strata (51.1% vs. 59.0% vs. 54.6%, respectively; *p* = 0.346). The overall mean was 24.9 (9.3) kg, with mean values of 24.0 (8.5) kg in the 3–9.9 mg/L group, 24.8 (9.2) kg in the 10–49.9 mg/L group, and 26.5 (10.9) kg in the ≥50 mg/L group.

The TUG test was successfully performed in 81.1% of the total sample at baseline. Stratified by CRP levels, the proportion of patients able to complete the test was significantly lower in the highest CRP group (62.8%) compared to those with CRP 3–9.9 mg/L (86.3%) and CRP 10–49.9 mg/L (84.9%) (*p* = 0.0016). Of those completing the TUG test, the average completion time was 14.5 s (SD 6.6), with no significant intergroup differences (*p* = 0.563). Specifically, mean times were 14.1 (6.5) seconds in the CRP 3–9.9 mg/L group, 15.0 (6.9) seconds in the 10–49.9 mg/L group, and 13.9 (6.3) seconds in the ≥50 mg/L group. When categorizing performance, 22.3% of patients overall were classified at risk (TUG > 20 s), with 18.3% in the CRP 3–9.9 mg/L group, 27.7% in the 10–49.9 mg/L group, and 15.6% in the ≥50 mg/L group (*p* = 0.213).

Regarding the chair stand test, it was performed by 73.6% of the total sample, with significant differences across CRP strata: 77.9% completion in the 3–9.9 mg/L group, 78.2% in the 10–49.9 mg/L group, and only 54.9% in the ≥50 mg/L group (*p* = 0.005). Among those who performed the test, the mean number of repetitions was 5.7 (2.9) overall, with no statistically significant differences between CRP categories (*p* = 0.077): 6.0 (3.2) in the 3–9.9 mg/L group, 5.1 (2.3) in the 10–49.9 mg/L group, and 6.2 (3.8) in the ≥50 mg/L group. When results were classified based on risk, 42.1% of participants showed impaired performance (fewer than five repetitions), with a distribution of 36.5% in the 3–9.9 mg/L group, 50.5% in the 10–49.9 mg/L group, and 28.6% in the ≥50 mg/L group (*p* = 0.059).

### 3.5 Biochemical and inflammatory markers

Biochemical parameters showed a clear inverse association with the degree of inflammation. Serum albumin concentrations progressively decreased across increasing CRP categories, while ferritin levels showed a significant upward trend. Inflammatory indices such as the CRP/albumin ratio, neutrophil/lymphocyte ratio, and platelet/lymphocyte ratio increased significantly in parallel with higher CRP levels. Full results are detailed in Table 3.

**TABLE 3:** Biochemical and inflammatory markers by CRP group.

		10–49.9 mg/L	≥50 mg/L	*P*-value
Albumin (g/dL)	3.90 (0.57)*	3.71 (0.72)#	3.35 (0.66)*#	<0.001
Prealbumin (mg/dL)	20.9 (8.1)*#	18.3 (7.2)#	19.3 (15.3)*#	0.502
CRP (mg/L)	5.4 (1.8)*	23.4 (10.7)*	103.9 (51.1)*	<0.001
Ferritin (ng/mL)	273.0 (382.5)*#	677.8 (1603.0)#	803.2 (955.4)*	0.031
CRP/prealbumin ratio	0.32 (0.24)*	4.48 (28.0)*	10.9 (16.4)*	0.026
CRP/albumin ratio	1.43 (0.51)*	7.15 (7.56)*	32.95 (18382)*	<0.001
Neutrophil/lymphocyte ratio	2.99 (3.11)*	4.15 (3.72)	5.14 (4.25)*	<0.001
Platelet/lymphocyte ratio	165.9 (114.2)*	212.0 (165.2)	266.8 (232.1)*	0.002
Systemic inflammtory index	780.6 (943.8)*#	1208.7 (1387.4)#	1604.6 (1660.7)*	0.001

Values are expressed as mean (standard deviation). CRP, C-reactive protein. For the comparison among groups *p < 0.001; #*p* < 0.05.

### 3.6 Quality of life

Quality of life assessment revealed significant differences only in the self-care dimension, with a higher proportion of patients reporting problems at increasing CRP levels. Detailed results are shown in Table 4.

**TABLE 4:** Quality of life parameters by CRP group.

		10–49.9 mg/L	≥50 mg/L	*P*-value
EQ-5D VAS	58.6 (19.5)	53.2 (22.1)	60.0 (19.6)	0.150
Time trade-off score	0.66 (0.26)	0.57 (0.28)	0.59 (0.31)	0.087
Mobility –% with problems	39.1	51.3	52.1	0.139
Self-care –% with problems	21.7	40.9	41.7	0.029
Usual activities –% with problems	45.6	57.4	58.3	0.449
Pain/discomfort –% with problems	60.9	65.2	62.5	0.703
Anxiety/depression –% with problems	62.0	60.9	60.4	0.969

Values are expressed as percentage of patients with moderate to extreme problems and as mean (standard deviation) scores on the EQ-5D-5L domains. VAS = visual analogue scale (0–100), where higher values indicate better self-perceived health. EQ-5D-5L scores range from 1 (no problems) to 3 (extreme problems).

### 3.7 Mortality

**FIGURE 1:**
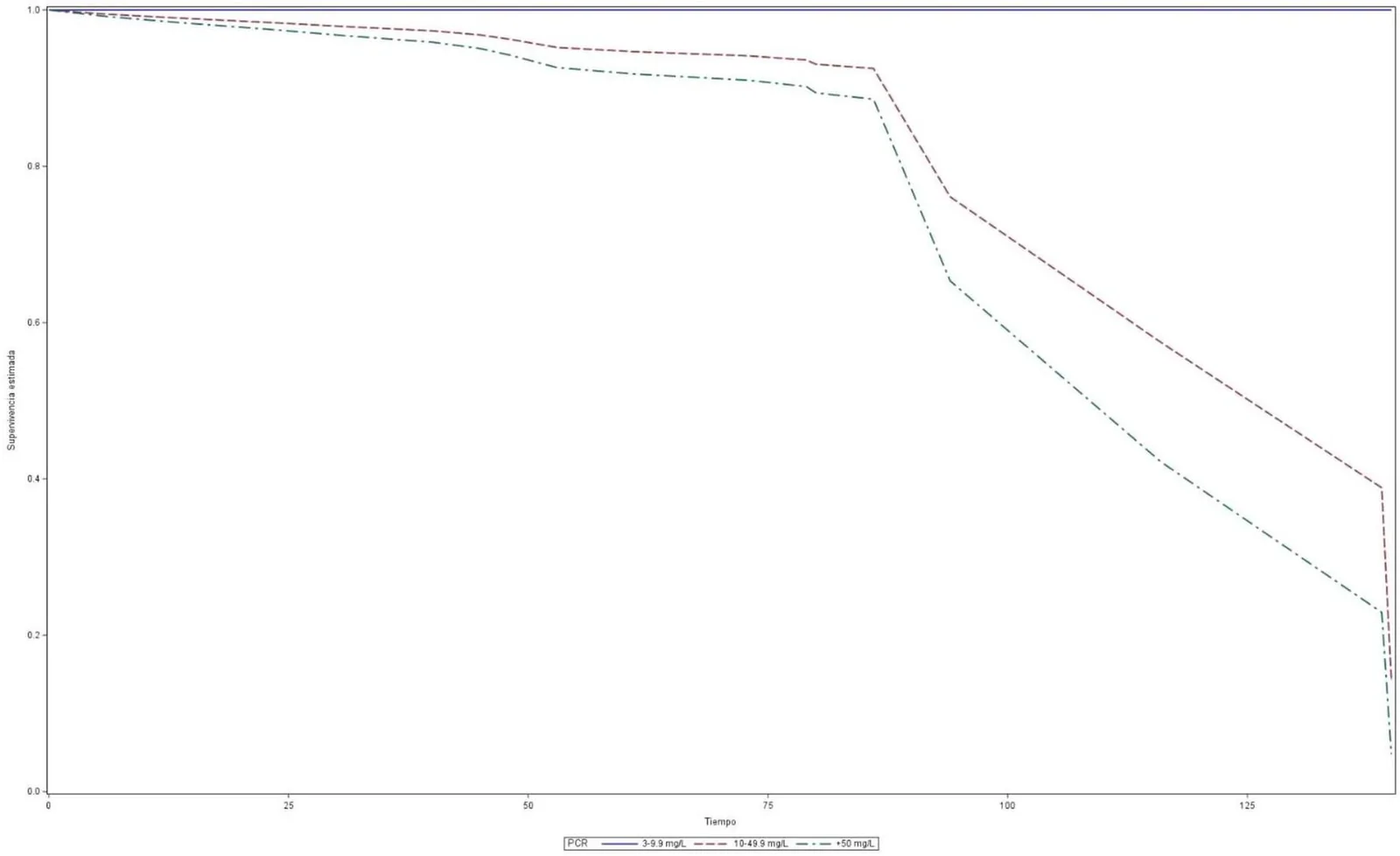
Kaplan-Meier survival curves stratified by CRP levels: Survival probabilities over 90 days of follow-up are shown for malnourished patients stratified by initial C-reactive protein (CRP) levels: low (3–9.9 mg/L), intermediate (10–49.9 mg/L), and high (≥50 mg/L). Patients with higher CRP levels exhibited reduced survival, while few deaths occurred in the low CRP group. The differences among survival curves were statistically significant (Log-rank test, *p* = 0.0087). Hazard ratios were estimated using Cox proportional hazards models adjusted for age, sex, malnutrition severity (GLIM stage), and handgrip strength.

At 3-months follow-up, overall mortality in the cohort was 9.1%. Mortality rates increased significantly with higher levels of baseline inflammation. Specifically, 1.1% of patients with CRP levels between 3 and 9.9 mg/L died during follow-up, compared to 11.8% in the 10–49.9 mg/L group and 17.7% in those with CRP ≥ 50 mg/L (*p* < 0.001). Compared to patients with CRP levels of 3–9.9 mg/L, those with CRP ≥ 50 mg/L had a significantly higher risk of mortality (OR 20.14, 95% CI 2.47–164.1, *p* < 0.05), as did those with CRP 10–49.9 mg/L (OR 12.53, 95% CI 1.62–97.1, *p* < 0.05). The Kaplan-Meier curves are presented in Figure 1. Using the combined categories of CRP 3–49.9 mg/L as the reference group, both crude and adjusted hazard ratios (HRs) indicated a non-significant increased risk of mortality associated with CRP levels ≥ 50 mg/L. The crude HR was 2.58 (95% CI: 0.95–7.00), while the adjusted HR, controlling for sex, age, sarcopenia status, and handgrip strength, was 2.09 (95% CI: 0.70–6.23).

## 4 Discussion

In this prospective multicenter cohort study, we observed that increasing levels of systemic inflammation, defined by CRP categories according to the GLIM-endorsed cut-offs, were associated with marked alterations in inflammatory and biochemical markers, impaired functional performance, and higher mortality in patients with disease-related malnutrition. Patients with severe inflammation (CRP ≥ 50 mg/L) showed significantly lower serum albumin and higher inflammatory indices, as well as reduced ability to complete functional tests such as the chair stand and Timed Up and Go, compared with those in lower CRP strata. 3-months mortality rose with higher CRP levels, from 1.1% in the lowest group to 17.7% among those with severe inflammation. To our knowledge, this is the first multicenter outpatient study directly applying GLIM-endorsed CRP thresholds to DRM stratification. Although crude analyses showed a significant increase in mortality across CRP categories, the association between CRP ≥ 50 mg/L and 3-months mortality did not reach statistical significance after adjustment for age, sex, malnutrition severity, and handgrip strength. Therefore, these findings should be interpreted with caution

Our study confirms that systemic inflammation, assessed through CRP thresholds proposed by the GLIM framework, is closely associated with reduced functional performance, and increased short-term mortality in ambulatory patients with disease-related malnutrition. These results align with the broader evidence demonstrating the prognostic value of CRP across clinical settings. Although most studies have been conducted in hospitalized or critically ill populations–where persistently high or rising CRP levels consistently predict adverse outcomes –emerging data in ambulatory cohorts support a similar relationship (12–14).

In patients undergoing chronic dialysis, elevated CRP independently predicts two- to 3-years mortality, both overall and from cardiovascular causes (15–17). Likewise, in elderly outpatients with sepsis, the combination of CRP, albumin, and mobility status predicts 28-days mortality with accuracy comparable to established clinical scores (18). Similar associations have been described in acute ischemic stroke, where higher CRP levels are linked to increased all-cause mortality in a dose-dependent manner, and in surgical populations, where pre- and postoperative CRP strongly correlates with mortality after hip fracture (19, 20). Taken together, these findings reinforce that CRP, alone or in combination with albumin, provides valuable prognostic information not only in acute hospital settings but also in ambulatory populations. Our results extend this evidence to patients with disease-related malnutrition, highlighting the utility of integrating CRP thresholds into outpatient nutritional assessment and follow-up.

The strong relationship observed between systemic inflammation and nutritional decline is supported by mechanistic evidence. Elevated CRP reflects a cytokine-driven acute phase response, during which hepatic protein synthesis is reprioritized–albumin production is downregulated by up to 50%, while acute-phase proteins such as CRP are markedly increased (21). Beyond hepatic effects, inflammation promotes muscle wasting through activation of the ubiquitin–proteasome and autophagy–lysosome systems, accelerating protein degradation and contributing to cachexia (22). Mitochondrial dysfunction further exacerbates this catabolic state, impairing cellular energy metabolism and favoring loss of lean tissue mass (23). Inflammation also reduces dietary intake by disrupting central appetite regulation. Elevated CRP levels ≥ 3 mg/dL have been linked to anorexia in geriatric cohorts, mediated through altered neuroendocrine signaling and increased production of appetite-suppressing cytokines (24). This bidirectional relationship establishes a vicious cycle: malnutrition exacerbates systemic inflammation, while inflammation worsens nutritional decline, perpetuating a downward spiral of frailty and functional impairment (25).

In ambulatory patients, elevated CRP has consistently been associated with poorer physical function across diverse clinical and community-based cohorts. In chronic obstructive pulmonary disease, raised CRP levels accompany increased interleukin-6 and impaired energy metabolism, leading to reduced performance on functional tests such as the 6-min walk and worse symptom scores (26). Among community-dwelling older adults, higher CRP concentrations are linked to slower walking speed, weaker handgrip strength, and poorer performance on composite physical function measures, such as the Short Physical Performance Battery and the Tinetti test (27–29). In disease-specific populations, CRP also predicts functional deterioration. In patients with chronic stable angina, higher CRP levels were associated with worse New York Heart Association functional class and reduced left ventricular ejection fraction (30). In early rheumatoid arthritis, persistent CRP elevation predicted worsening disability scores, while normalization or reduction of CRP was linked to functional improvement (31). Furthermore, in middle-aged African Americans, elevated CRP correlated with poorer scores in the Short Physical Performance Battery, greater disability in activities of daily living, and lower lean body mass percentage. Taken together, these findings reinforce the notion that CRP is not merely a passive biomarker of inflammation but also a surrogate of impaired energy metabolism and muscle strength, reflecting systemic processes that compromise functional reserve. This evidence is consistent with our observation that higher CRP levels in ambulatory patients with disease-related malnutrition were associated with reduced functional capacity and increased short-term mortality, even though they did not consistently correlate with differences in body composition by ultrasound or bioimpedance.

This study presents several methodological strengths. First, the prospective, multicenter cohort design allows for a clear temporal sequence between exposure (inflammatory status, assessed by CRP levels) and clinical outcomes. The inclusion of diverse clinical nutrition units across Spanish hospitals enhances external validity and generalizability within hospital-based populations. Moreover, the application of strict inclusion criteria, including confirmation of disease-related malnutrition (DRM) based on the GLIM framework and a CRP threshold > 3 mg/L, ensured a well-defined and homogenous study population with confirmed inflammatory status. Another major strength lies in the comprehensive assessment of nutritional status using objective and validated methods such as anthropometry, single-frequency bioelectrical impedance analysis (BIA), and nutritional ultrasound^®^. Functional capacity was evaluated through standardized physical performance tests, and quality of life was assessed using the EQ-5D-5L instrument, thereby providing a multidimensional view of patient health. Furthermore, the analysis incorporated several inflammation-related biomarkers and indices (e.g., CRP/albumin ratio, neutrophil-to-lymphocyte ratio), strengthening the reliability of the inflammatory classification beyond CRP alone.

However, the study also has some limitations. Notably, the absence of a comparator without systemic inflammation limits the ability to evaluate the independent effect of inflammation on nutritional and clinical outcomes. The exclusive inclusion of patients with CRP > 3 mg/L may restrict generalizability to the broader population of patients with DRM and minimal or no inflammatory response. Additionally, the 3-months follow-up period, while sufficient to capture short-term mortality, may be inadequate for assessing longer-term functional or nutritional recovery. The relatively small number of mortality events (*n* = 24) may have limited statistical power for multivariable survival analyses. Therefore, the non-significant adjusted hazard ratios should be interpreted with caution, and larger prospective cohorts are warranted to confirm these findings. Although the AFEDIN study was conceived as a prospective multicenter cohort, the present analysis is predominantly cross-sectional, as nutritional status, inflammatory markers, functional performance, and quality-of-life measures were assessed only at baseline, with 3-months mortality as the sole longitudinal outcome. Consequently, the associations observed between CRP levels and nutritional, functional, or quality-of-life parameters should be interpreted as concurrent relationships rather than as evidence of temporal or causal effects. This design precludes determining whether systemic inflammation precedes functional decline and impaired quality of life, or whether these alterations coexist as manifestations of the same underlying disease burden. Future studies should incorporate repeated measurements of GLIM phenotypic and etiologic criteria, inflammatory biomarkers, physical function tests, and quality-of-life instruments over time. Such longitudinal designs would allow fully prospective analyses, enable evaluation of trajectories and causal pathways, and clarify the dynamic interplay between inflammation, disease-related malnutrition, functional deterioration, and patient-centered outcomes.

In this prospective multicenter study of patients with disease-related malnutrition, systemic inflammation–quantified by CRP levels using GLIM-endorsed thresholds–was strongly associated with impaired functional capacity, increased self-care difficulties, and higher short-term mortality, even in the absence of marked differences in body composition. These findings highlight CRP as a clinically meaningful prognostic biomarker, reinforcing its role within the GLIM framework. Incorporating objective inflammatory markers into routine nutritional assessment can improve risk stratification, support individualized treatment strategies, and guide timely interventions to prevent functional decline and adverse outcomes in malnourished patients.

## Data Availability

The raw data supporting the conclusions of this article will be made available by the authors, without undue reservation.

## Footnotes

1
www.ienva.org
